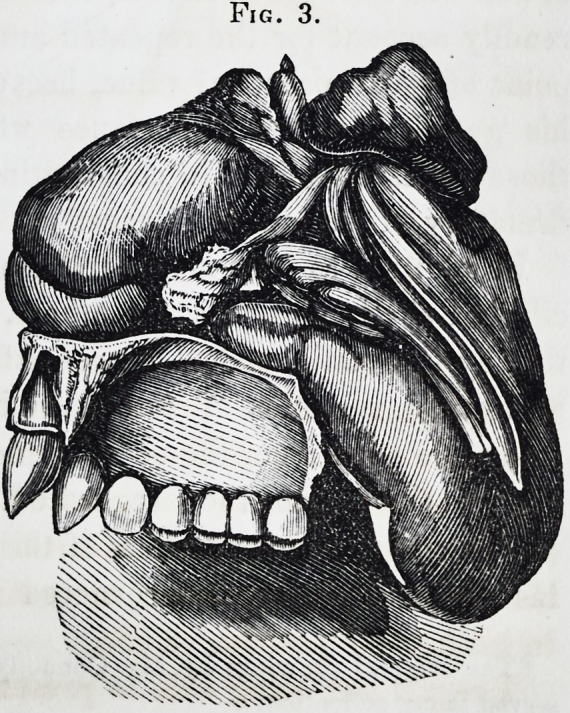# On Extirpation of the Entire Upper Jaw, Illustrated by a Case in Which It Was Removed, Together with the Whole of the Palate Bone, on Account of a Large Fibro-Vascular Tumor Springing from the Antrum

**Published:** 1855-04

**Authors:** Richard G. H. Butcher

**Affiliations:** Surgeon to Mercer's Hospital, Member of Council of the Royal College of Surgeons in Ireland, and Examiner on Anatomy, Physiology, and Pathology thereto for the past five years, &c. &c.


### SELECTED ARTICLES.

### ARTICLE XV.

*On Extirpation of the entire Upper Jaw, illustrated by a Case in which it was Removed, together with the whole of the Palate Bone, on account of a large Fibro-vascular Tumor springing from the Antrum.* By Richard G. H. Butcher, F. R. C. S. I. Surgeon to Mercer's Hospital, Member of Council of the Royal College of Surgeons in Ireland, and Examiner on Anatomy, Physiology, and Pathology thereto for the past five years, &c. &c.

There are certain forms of disease affecting the cavity of the antrum, as well as others springing from its walls, and involving the osseous tissue, which call for the operative interference of the surgeon, and demand the removal of the upper jaw, either in part or in its totality. I allude more particularly to various morbid growths and tumors, characterized as fibrous, cartilaginous, encephaloid, fatty, erectile, or osseous, in their nature. It is true, that all do not run to a fatal issue with the same rapidity; but it is equally certain that the most benign in character may, by enlargement, produce such an impairment of, and pressure upon, the surrounding organs and textures as must ultimately prove fatal.

The operations on the upper jaw may, in reality, be classed under two heads—that of the exsection and that of the disarticulation of the bone; and this I conceive to be a wholesome division of the subject, for by it may be cleared up the discrepancy which prevails amongst writers, relative to the priority in this operation claimed by illustrious surgeons.

Years ago, so far back even as 1693, a part of the upper jaw was removed by Akoluthus, a physician at Breslau. He being consulted by a woman who had a tumor on the jaw which followed the extraction of a tooth, enlarged the mouth with a cut, removed part of the swelling, together with four teeth, but not being able at once to get completely round it, he attacked it several times at intervals of a few days, sometimes with cutting instruments, and sometimes with the actual cautery, and at last succeeded in curing his patient.*

Planque mentions a case of sarcoma, the size of two fists, in which the cheek was divided to excise the tumor, and two or three teeth, and a portion of the corresponding bone, were removed with it. The patient recovered.

The scooping operation was likewise put in practice by Desault, Garengeot, Jourdain and others, and has been in modern times more especially brought under notice by Dupuytren, in 1820, and since then by many surgeons. The latter distinguished pathologist even went further, and argued that the greater part of the jaw might be excised; and he was induced to form this opinion from the consideration of the examples on record, where the patient recovered after most severe mechanical injuries of the face, and necrosis, occasioning the destruction of the bone. A most remarkable instance, where the jaw came away almost entire in this latter affection, is mentioned by Camper, and the patient survived. Dupuytren, though he did not

*Mémoires de l'Académie Royale de Chirurgie, tom. v. 1819.

remove the entire bone on the date above mentioned, at all events cut out the greater part of it in 1824; yet, too, in the practicability of this operation he was anticipated by Desault and Bidloo, who, however, confined themselves to recommending, without ever having, as it appears, performed it. That Dupuytren did not remove the whole jaw, or, in other words, disarticulate it, is proved by Gensoul, who says:—"I saw the two practitioners, Sanson and Pinel Grandchamp, who were stated to have witnessed these operations, for the purpose of knowing what method had really been adopted. Sanson informed me that he had no knowledge of the fact of an entire removal of the superior maxillary bone, and that he knew only of the operation performed in 1820, which was similar to Desault's, and of one other in the year 1824; and that in the latter case a large piece of the edge of the alveolar process had been removed with a small saw. Pinel Grandchamp said he had witnessed the two operations mentioned by Sanson, but he had never heard say that Dupuytren had ever thought of removing the whole superior maxillary bone."* M. Pillet corroborates this testimony, for he states, the patient last operated on by Dupuytren died at Salpetrière, and on examination a portion of the jaw was found left behind.†

The nearest approach to the removal of the whole superior maxillary bone is detailed in "White's Cases in Surgery." It was operated on successfully by his father, and he relates it in the following manner:—"The patient was a woman, afflicted with a tumor betwixt the zygomatic process and the nose, arising from the lower part of the orbit of the left eye. It pressed the nostrils to one side, so as to stop the passage of the air through them, and thrust the eye out of its orbit, so that it lay on the left temple, yet, though thus distorted, it still performed its office. The tumor occupied the greater part of the left side of the face, extending from the lower part of the upper jaw to the top of the forehead, and from the farthest part of the

*Lettre Chirurgicale sur quelques Maladies graves du Sinus Maxillaire et de l'Os Maxillaire Inférieure. Paris, 1853.

†Lancette Française, tom. ii, p. 284.

left temple to the external canthus of the eye. It had an unusual and equal bony hardness. It was of a dusky livid color, with varicose veins on the surface, and there was a soft tubercle projecting near the nose, where nature had endeavored, in vain, to relieve herself." For the removal of the disease, he continues:—"I began with a semicircular incision below the dislocated eye, in order to preserve that organ, and as much as possible of the orbicular muscle; then carrying the incision round the external part of the tumor, I brought it to the bottom of it, and then ascended to the place where I began, taking care not to injure the left wing of the nose. After taking away the external part of the tumor, which was separated in the middle, by an imperfect suppuration, there appeared a large quantity of matter, like rotten cheese, in part covered by a bony substance, which, however, was so carious as to be easily broken through. I scooped away abundance of this matter, with a great many fragments of rotten bones. Upon cleansing the wound from blood and filth with a sponge, I found the left bone of the nose and the zygomatic process carious, and easily removed them with an elevator. There were no remains of the bones composing the orbit of the eye, which were plainly destroyed by the same disease. The optic nerve was denuded as far as the dura mater; and the dura mater and pulsation of the vessels of the brain were apparent to the eye and touch. The left superior maxillary bone, in the sinus of which this disease had its origin, and remained a long time concealed, was surprisingly distended, and in some places became carious; it had exfoliated from the lower part to the sockets of the teeth, which part was, in like manner removed. I applied the actual cautery to the rest of the bones and putrefied parts, taking care not to injure the eye and neighboring parts which were sound. The patient drew her breath through the wound, and was so incommoded by the fetid matter flowing into her throat, that she was obliged, for several weeks, to lie on her face to prevent suffocation. . . . The patient recovered, the eye returned to its place, and she enjoyed the perfect sight of it."*

* Page 135, *et seq.*

There can be no doubt that Mr. Lizars, of Edinburgh, is justly entitled to the credit of having, in 1826, proposed the *entire removal* of the superior maxillary bone, and of explaining the proceedings for its accomplishment. Speaking of "polypi, or sarcomatous tumors, which grow in the antrum," he says: "All the cases which have come within my knowledge (with the exception of one) wherein these sarcomatous tumors have been removed by laying open the antrum, have either returned or terminated fatally. I am, therefore, decidedly of opinion, that unless we remove the whole diseased surface, which can only be done by taking away the entire superior maxillary bone, we merely tamper with the disease, put our patient to excruciating suffering, and ultimately to death. The inferior maxillary bone has now been nearly entirely removed for osteosarcoma with success, and I see no difficulty in accomplishing the same with one of the superior maxillary. We secure the common carotid artery for other tumors of the face, and for aneurism by anastomosis, and why not do it for so loathsome and fatal a disease as this? The steps or plan of the operation I would suggest for so fatal a disease are—first, to secure the trunk of the common carotid artery of the affected side; next, to make an incison through the cheek, from the angle of the mouth backwards or inwards, to the masseter muscle, carefully avoiding the parotid duct, then to divide the lining membrane of the mouth, and to separate the soft parts from the bone, upwards, to the floor of the orbit; thirdly, to detach the half of the velum palati from the palate bone. Having thus divested the bone to be removed of its soft coverings, the mesial incision tooth of the affected side is to be removed; then the one superior maxillary bone to be separated from the other, at the mystachial and longitudinal palatine sutures, and also the one palate bone from the other at the same palatine suture, as the latter bone will also require to be removed either by the forceps of Mr. Linton or a saw; thirdly, the nasal process of the superior maxillary bone should be cut across with the forceps; fourthly, its malar process, where it joins the cheek bone; fifthly, the eye, with its muscles and cellular cushion, being carefully held up with a spatula, the floor of the orbit is to be cleared of its soft connections, and the superior maxillary bone separated from the lachrymal and ethmoid bones with a strong scalpel. The only objects now holding the diseased mass are, the pterygoid processes of the spheroid bone, with the pterygoid muscles. These bony processes will readily yield by depressing or shaking the anterior part of the bone, or they may be divided by the forceps, and the muscles cut with the knife. After the bone with its diseased tumor has been removed, the flap is to be carefully replaced, and the wound in the cheek held together by one or two stitches, adhesive plaster, and bandage. In no other way do I see that this formidable disease can be eradicated."* The foregoing operation, which Lizars proposed, he made the attempt to perform in December, 1827, and he thus mentions it:—"I attempted to remove the bone for a medullary sarcomatous tumor of the antrum, from a miner or collier, after securing the common carotid artery of the affected side, but I was prevented by the hemorrhagic disposition of the gum and palate, my patient having lost in a few seconds upwards of two pounds of blood, which welled out at every incision as if there had been an aneurism by anastomosis. The man survived this attempt seventeen months."†

Though Lizars first proposed the operation, M. Gensoul, Surgeon to the Hotel Dieu, at Lyons, was the first to perform it. He removed every part of the superior maxillary bone, together with the whole of the palate bone, from a boy aged 17, on the 26th of May, 1827, for a large fibro-cartilaginous tumor, "occupying the whole left side of the face, and pushing to one side the orifice of the mouth; it extended from above downwards from the floor of the orbit to two lines above the chin, from before backwards, from the nose, which was thrust to the right, to the top of the angle of the inferior maxillary bone." Gensoul states, he was not aware what method Lizars had recommended,‡ but was induced to operate for the follow-

* A System of Anatomical Plates, &c., part xx—The Organs of Sense, 1826.

† Lancet, 1829-30.

‡ Lettres Chirurgicales sur quelques Maladies graves du Sinus Maxillaire, &c. p. 18.

ing reasons:—For several years previously he had known patients die of very tedious operations, undertaken for the removal of cancerous and other tumors of the antrum. Reflecting on the fate of these unfortunate individuals, he was led to conclude that others, laboring under similar diseases, might be cured by an operation, which consisted in freely denuding the antrum and upper jaw-bone, so as to be able to divide the sound parts, instead of meddling with the diseased ones, and of searching for the precise limits of the disease in the midst of blood and the remains of the affected textures. In short, he was induced to think, that the same principle should be acted upon in this operation, as is followed in others, undertaken for the extirpation of cancerous tumors in general. In this remarkable case, Gensoul did not first tie the carotid artery, as advised by Lizars, but made a vertical cut, from the inner corner of the eye directly down through the upper lip, opposite the left cuspid tooth. From the middle of this cut, or, rather, nearly on a level with the floor of the nose, he made a second, up to four lines from the front of the lobe of the ear, and a third cut, beginning five or six lines to the outside of the orbit down to the end of the second and third cuts, and then turned the flap up to the forehead. But, for the purpose of completely exposing the tumor, he was obliged to continue, from the junction of the second and third cuts, another along the inner edge of the masseter muscle, to within an inch of the base of the lower jaw, and this lower flap he turned down. He then commenced with a chisel and mallet, cutting through the outer margin of the orbit, near the suture connecting the malar and frontal bones, into the spheno-maxillary fissure; and next, cut through the zygomatic process of the malar bone. The maxillary bone being thus freed externally, he placed a very broad chisel below the inner angle of the eye, and carried it through the lachrymal bone and the orbital plate of the ethmoid; and in the same way detached the corresponding part of the nasal bone. Cutting away, with a bistoury, all the soft parts connecting the wing of the nose to the upper jaw, he proceeded to separate the two superior maxillary bones, which he effected easily and quickly, having drawn the first left incisive tooth, by introducing a chisel, not directly from before backwards, but by wriggling it through the mouth. Lastly, to detach the maxillary bone from the pterygoid processes of the sphenoid, and to destroy any connections with the back of the ethmoid still remaining, he thrust the chisel into the tumor, passing it obliquely in the orbit, so as to cut through the superior maxillary nerve which he was anxious not to drag, and pushing it sufficiently deep to form a lever, so that he could turn the tumor down into the mouth. This answered very well, and he had then only to divide, with curved scissors and bistoury, the attachments of the bone to the soft palate, so as to leave the latter unharmed. The operation was scarcely concluded when the patient fainted, but revived on being laid upon his bed. This patient perfectly recovered.

Lizars, though being disappointed in carrying out his views in 1827, yet was not discomfited, and on August 1st, 1829, he performed his second operation. He first tied the trunk of the temporal and internal maxillary arteries, and also the external jugular vein, which had been divided in the first incision. He cut through the alveolar process and bony palate on the left side of the palatine suture, and completely separated the upper jaw with the saw, Liston's forceps, and strong scissors, but the orbital plate was separated from the eyeball by the handle of a knife. The tumor was medullary sarcomatous, and a portion of it, attached to the pterygoid process of the sphenoid bone, could not be detached, but part of the malar bone involved in the disease was removed. On the sixteenth day the wound had healed, and she left the house on that day. Three days after, she expired suddenly, but no examination was permitted."* Lizars' third and successful operation was performed on January 10th, 1830, on a woman, the external carotid artery having been first tied. After slitting up the nostril, making a flap of the cheek, and divesting the bone of its coverings where it was to be sawn through, he applied the saw on "the front of the superior max-

* London Medical Gazette, vol. v. p. 92.

illary bone, between the nostril and the mouth, or at the side of the mystachial suture; on the palatine plate backwards from this, parallel with the longitudinal palatine suture, to near where the transverse palatine suture exists; across the same palatine plate towards the bulbous process upwards, between the bulbous process and the pterygoid processes of the sphenoid bone, across where it joins the cheek bone; and, lastly, at its nasal process, parallel with the inferior margins of the lachrymal and nasal bones. I then, with strong scissors, cut the connections of the orbitary process of the palate bone deep into the orbit, to the spheno-maxillary fissure, and was lastly able, by notching with the bone forceps at every point where the saw had been, to remove the entire bone, which had its cavity filled with a firm sarcomatous tumor. The patient was able to walk about her room on the eighth day, and went out to take an airing on the thirtieth day; and she left the hospital on the 5th of March following."*

From a dispassionate consideration of the subject, I have no doubt Mr. Lizars is to be regarded as the originator of this operation, the propriety of performing which, in certain cases, is abundantly borne testimony to by the experience of modern times; and in conjunction with his name I would associate that of the illustrious Liston, to whom we are indebted for a discrimination of the cases in which the operation may with propriety be undertaken.† Indeed, in this special department of our art, he has, in a marked way, left the impress of both his labor and his genius. To the list of names already mentioned, Lizars, Dupuytren, Gensoul, Liston, and White, we may likewise add those of Syme, Robert, Mott, Velpeau, Lisfranc, Dieffenbach, O'Shaughnessy, Heyfelder, Fergusson, and Cusack, as most closely associated with this bold procedure of modern surgery, and prominently conspicuous in its advocacy, both by precept and by practice. The following case, in which I extirpated the entire of the upper jaw, together with the whole of the palate

* Lancet, 1829–30.

† On Tumors of the Mouth and Jaws. Medico Chirurgical Transaction, vol. xx.

bone, adds another to the list of those on record which have been successful.

Patrick Higgins, aged sixteen years and six months, was admitted into Mercer's Hospital, under my care, March 1, 1853. The patient stated that a polypus had been extracted from his right nostril nine months before admission, but as to the time of its commencement he was entirely ignorant; he could only affirm with confidence, that for three months before the operation the tumor projected into the nose, and interfered with free respiration. He then applied to a surgeon, who removed the growth with the ordinary polypus forceps: during the time of extraction, and afterwards, there was profuse bleeding. The morbid growth had been removed scarcely a month when it again appeared, and manifested a more rapid tendency to increase, which gradually progressed up to the date of his admission. On the closest interrogation, little information could be obtained as to the earlier symptoms that ushered in the local affection. True it is, the patient could remember the presence of a dull, aching pain constantly fixed over the right brow, and of a more acute, severe, and lancinating character beneath the under eyelid. These symptoms, together with the blocking up of the nose, were the inconveniences which urged him to seek for surgical advice, when the operation referred to was put into execution with temporary relief. On the re-growth of the tumor, the patient was brought a considerable distance from the country, and placed under my care. At this time his condition was exactly as follows:—The features were greatly distorted; the right superior maxillary bone being rendered prominent by expansion of its walls, owing to pressure exerted from within; the soft parts comprising the cheek were not discolored, yet considerably thinned and rendered tense over the projecting part; handling the tumor, and even pressure, did not elicit pain, and there was total absence of all œdema. The symmetry of the eye was lost, the lower lid being raised above the level of that upon the sound side, and the entire organ was somewhat elevated from its bed by the displacement upwards of the floor of the orbit; the lachrymal secretion was profuse, the nasal duct obstructed, and, as a sequence, the tears constantly flowed over the cheek; the nose was expanded and pushed to the left side—a derangement which will partly account for the eyelid not descending properly; a large, fleshy mass occupied the right nostril, and filled the cavity to its external margin; this anterior part of the tumor was of a dark color, and coated with a thin crust, yielding in abundance a thin, ichorous discharge; it was soft, elastic, and bled on the slightest touch; and so forcibly was pressure exerted by the growth of the tumor towards the mesial line, it was sufficient to destroy the bony septum, and block up the left nostril also; the cartilage constituting the anterior part of the partition being spared, doubtless owing to its elasticity, was forced over, and by the bulk of the growth retained in apposition with the left ala, so as to occlude the anterior aperture of the left nasal cavity also; a probe could be made to traverse in an arch over the superior surface of the tumor, but was instantly stopped, both internally and externally—externally, by the growth protruding from the antrum, and internally or mesially by the irregularity of the surface. By careful manipulation a bent probe could be insinuated beneath the tumor and carried along the floor of the right nostril, and made to appear below the edge of the soft palate. Within the mouth, the tumor could be detected taking a backward direction, pressing down the entire hard palate, and projecting into the pharynx, forcing the velum downwards and forwards, thus placing it almost vertically; in this acquired position the growth could be distinctly felt, and on palpitation was obviously elastic; there was no pain occasioned by the handling of it, though the mucous membrane over the entire region was preternaturally colored, presenting, in many points, patches of ramiform vascularity; the alveolar ridge was perfect, and the teeth were not loosened; respiration and deglutition were considerably interfered with, owing to the obstruction in the nostrils and the projecting mass in the pharynx.

From a very careful inspection and consideration of the case, I at once came to the conclusion that no temporizing measures would avail, because the jaw bone itself was implicated, and the tumor had its root and origin within the antrum, filled the cavity, expanded its osseous walls, and, finally, burst from its confinement, and threatened life by an interruption of functions essential to existence ; therefore the removal of the upper jaw in its totality promised the only chance of permanent relief. The patient had been in hospital only a few days when a rapid change in his condition urgently called for operative interference. The tumor suddenly increased with almost incredible rapidity ; all its surfaces seemed to participate in the enlargement, but posteriorly the change was most alarmingly felt ; the tumor was now plainly visible below the soft palate, and so far interfered with the respiration that the sufferer could not lie down without experiencing a sensation of smothering, or obtain any sleep without being almost instantly awakened by a feeling of suffocation and painful gasping for breath. The power of deglutition was likewise interrupted ; fluids could only be swallowed, and that very sparingly, even in sups. My proposal to extirpate the jaw was acquiesced in by my colleagues, Messrs. Tagert, Jameson, and Bevan, and sanctioned by the high authority of Mr. Cusack. The operation was performed in the following manner, March the 5th, 1853 :—

The patient being seated on a chair, with his head resting on the breast of an assistant, I passed a strong, curved bistoury, guarded on my finger, into the mouth, thrusting out the point a little external to the junction of the malar and maxillary bones on the right side, and slit the cheek from this point downwards to about a few lines in front of the angle of the mouth ; I then, with a scalpel, continued the incision from the point where the bistoury first appeared, a short way upwards and outwards. The knife was next applied half an inch below the inner canthus of the eye, over the nasal process of the maxilla, and carried down at the side of the nose, round the ala, and then straight through the upper lip. The flap thus formed was rapidly dissected up from its attachments and held by an assistant, the orbital edge of the maxilla was cleared from the soft parts, and the attachment of the inferior oblique muscle accomplished ; thus it, together with the nasal process and the anterior part of the malar bone, lay fully exposed. At this stage, the flow of blood was so profuse it was considered advisable to secure the facial artery at the point where it was divided in the outer incision, and likewise the transverse facial on the same side: the facial artery on the left side was commanded by pressure, where it passes round the jaw in front of the masseter muscle. The cartilage of the ala was next detached from the bone, and the nose drawn over to the left side. The division of the osseous structure was next accomplished, by means of a powerful scissors: the malar bone, at its junction with the maxilla, was first cut through; then the nasal process of the maxilla divided; next, the first incisor tooth being drawn, one blade of the scissors was passed into the nostril on the affected side, the other into the mouth, and the palate plate severed, through its entire extent, from its fellow of the opposite side; the incision passed in a straight line from the gap occasioned by the extraction of the tooth to the posterior edge of the palate plate of the palate bone, where the tumor lay in contact with it. I then, with a strong, curved scissors, cut across the orbital plate, from the orbital margin of the maxillary bone, leaving a small portion of the floor of the orbit perfect, and by a careful stroke of the knife detached the velum pendulum from the palate bone. The maxilla was next grasped with a strong forceps, and forcibly pressed, so as to break down its connections behind, and make the tumor start from its bed; thus the entire mass was depressed, and drawn forwards by the aid of the forceps held in the left hand, while, with the index finger of the right passed in above the tumor, extensive adhesions were torn through; and, by a few additional touches of the knife, the entire mass was liberated from its attachments and taken away. The mouth was next sponged out, and very carefully examined: not a portion of the tumor remaining behind. The hemorrhage after was very inconsiderable, and yielded to the pressure exerted by small pieces of dry sponge thrust against the surface, each being guarded with lint and a string; the former to prevent the granulations shooting into its structure, and the latter to facilitate removal. Additional pieces of lint were introduced, so as to fill the cavity, and prevent the cheek sinking too much in. The patient was then placed in a recumbent position; nevertheless, syncope supervened, in consequence of the shock and loss of blood necessarily resulting from so formidable an operation. However, by the application of ammonia to the nose, the admission of fresh air, and the administration of wine, this faintness passed away, and the heart and brain resumed their functions. After this pause I proceeded to dress the wounds, by most carefully adjusting the cut surfaces, and retaining them in apposition by several points of the twisted and interrupted suture: thus was the dressing completed—neither pledgets, plasters, nor bandages being had recourse to. Immediately afterwards, the patient was removed into a private ward, put to bed, and a warm anodyne administered.

3 P. M.—The patient had sound sleep since morning—the fatigue, anxiety, and restlessness which he endured for two nights before the operation will partly account for the occurrence—and he took, with appetite, a quantity of boiled milk, and eggs beaten up in it, for nourishment.

March 6th.—Sleep was procured at intervals during the night. He suffers but little pain. Pulse quiet and compressible; skin soft; urine passed in considerable quantity; face but little swollen, and neither uneasiness nor tension in the site of the sutures. The patient was made to lie upon his left side, so that the profuse salivation might escape without interrupting the adhesion of the cut parts. Boiled milk and eggs to be liberally supplied, and also a pint of strong beef-tea.

2 P. M.—Pulse quickened; general restlessness; skin hot; symptoms evidently ushering in irritation. Ordered small doses of a sedative mixture, containing camphor julep, morphia, and prussic acid.

9 P. M.—Pulse lowered considerably, and quiet; refreshing sleep has been obtained for a short time. Ordered to continue the mixture at longer intervals; and to be allowed warm milk for drink through the night.

March 7th.—Slept almost uninterruptedly for the entire night. Pulse 90, and soft; skin cool. The wounds in the cheek seem united; there is but little swelling in their course, and the sutures are quite unproductive of irritation. The eye is perfectly on a level with the sound one, and the lid has resumed its proper position. There is slight fetor of the breath, occasioned by the presence of the plugs at the back of the pharynx (there now for forty-eight hours,) which called for their removal; this was easily effected by the injection of a little tepid water and gentle traction.

9 P. M.—Free from pain, and inclined to sleep.

March 8th.—The patient slept composedly during the early part of the night, and at intervals towards morning. Pulse quiet. Complains of a slight headache; face a good deal swollen, but no undue traction on the sutures; bowels confined. Ordered immediately a full emollient enema. I carefully sponged out the pharynx and mouth with a wash, containing chloride of soda. Eggs, beef-tea, &c., for nourishment. Salivation still very profuse.

9 P. M.—Patient slept nearly the entire day, and is free from pain.

March 9th.—The young man feels much refreshed after the night, and is most anxious for solid food; he can speak sufficiently distinct to be understood and make known his wants. His request I did not think it prudent to comply with, least any portion of the solid matter might become entangled in the irregular surface behind, and so bring on a fit of coughing, and thus tear asunder the recently united parts. The wounds of the face are all united by the first intention, nevertheless, the sutures being unproductive of irritation were suffered to remain. Eggs, milk, beef-tea, &c., for nourishment as before.

March 10th.—Removed all the sutures, and supported the parts with broad strips of adhesive plaster, the surface of the chasm left after the removal of the bone granulating healthily.

March 15th.—The patient was allowed to take solid food for the first time this day. The incisions in the cheek are not only united, but quite pale and nearly obliterated, and the surface on the interior is rapidly healing. On this day he was permitted to get up.

March 18th.—There has been a rapid amendment in the young man's condition: his speech and power of deglutition are greatly improved, and the salivation is considerably diminished. Brushed over the interior of the mouth and granulating surface with a solution of nitrate of silver, ten grains to the ounce.

It is unnecessary to follow up the daily report of the case any further; suffice it to say, that in a short time the parts were entirely healed, and he was dismissed perfectly cured.

Fig. 1.

At the time in which I write (May the 24th) the patient is in excellent health. Very little deformity marks the severe operation which had been performed: the gap left by the removal of the maxilla has contracted remarkably in size; the power of swallowing fluids and solids is naturally restored; and his speech and articulation are sufficiently distinct for a person in his humble position in life.

I regret not being able to present the reader with a drawing of the patient before the operation. The case so unexpectedly demanded immediate interference, time was not permitted for its execution, but the accompanying engravings. Figs. 2 and 3 exhibit a full sized representation of the parts removed. Fig. 1 is taken from a portrait which was drawn six weeks after the operation, attesting to the fact of the little amount of deformity consequent upon it.

FIG. 2.

FIG. 3.

*Examination of the part after removal.*—The structure of the tumor presented many interesting peculiarities. Its attachment and origin sprung from the outer part of the antrum. Not only was it incorporated with the lining membrane, but it likewise implicated the osseous wall. The surface from which it sprung, in the recent state, was softened, vascular, and pulpy; the upper surface of the tumor was lobulated where it encroached upon the orbit and elevated its floor; the lobules were of various sizes—some very small, but each consistent in structure, and invested by a dense capsule in a similar way to the larger masses of the growth. The entire tumor was remarkable for its great vascularity, which was more particularly confined to the posterior and upper surface; while on section the structure was dense by comparison, pale, eminently firm, and partaking of a fibrous, matted nature. This integral arrangement was very manifest under close examination with the microscope, and cleared away the suspicion which, on superficial inspection, might have been created of encephaloid disease being the synonyme most applicable to the growth. There was a total absence of all nucleated cells, either globular, caudate, or spindle-shaped; and above all, the section of any part only yielded a minute quantity of serum or blood on pressure, and not the true succus of cancerous tissue. The tumor, though destructive to the neighboring parts by pressure, yet did not appropriate or incorporate them in its structure. This peculiarity of non-malignant growth was strikingly manifest in the present instance; for, by pressure, producing interstitial absorption, the cancellated structure of the ethmoid and inferior spongy bones was attenuated and removed; and by the same process the vomer was detached from its position—a few shreds of it being spared and hanging loosely on the sinistral surface of the tumor. The vascularity of the growth, though remarkable on the surface, yet did not permeate its texture; hence a tendency to degenerate by assumed depravity of action was lessened. Again, the vascularity of the surface will readily account for the repeated and profuse losses of blood, a point of great practical value, because placing the surgeon on his guard as to the importance which should be attached to those repeated losses in constituting a diagnostic feature confirmatory of malignant disease.*

In addition to the modes of removing the jaw already specified, I shall mention a few others, practically put into execution by the distinguished names attached to them. Heyfelder performed resection of *both* jaws in the following way:—He made two cuts from the outer angles of the eyes into the corners of the mouth, then separated all the soft parts from the swelling to the inner corners of the eyes and to the nose bones. He next raised this four-cornered flap upon the forehead, car-

*The original drawings by Mr. Connolly, and the part removed, are preserved in my collection.

ried Jeffray's chain-saw through the proper fissure of the left orbit, and divided the connection of the left upper jaw bone and cheek bone. In like manner, he proceeded with the division of this bone from its connection with the frontal, lachrymal, ethmoid and nasal bones. In the same way, the right upper jaw bone was separated from its connections, and afterwards the vomer, and the still remaining connections were cut through with strong scissors. A lever-like pressure was made on the upper part of the tumor to complete the operation. Torsion and compression staunched the bleeding, and twenty-six sutures united the wound.* It is likewise stated by M. Velpeau that Mr. Rogers, of New York, removed both upper jaw bones as far back as the pterygoid processes.

Syme's directions for performing removal of the upper jaw bone are very simple, and will not be found less convenient than any other. He says: "Two incisions should be made through the cheek—one extending from the inner angle of the eye, directly downwards to the lip; the other beginning over the junction of the maxillary and malar bones, and terminating at the angle of the mouth. The triangular flap thus formed is to be dissected from the tumor, and the margin of the orbit exposed."† He then directs that "one blade of a large cutting pliers be introduced into the nose, and the other into the orbit, so as to divide the nasal process of the superior maxillary bone. The connection with the malar bone is next separated in the same way, and then the palate, previous to which one of the incisor teeth must be extracted if necessary. The surgeon having now deprived the bone of all its principal attachments, wrenches it out, either with his hands or strong forceps." Professor Syme afterwards abandoned this method by the two incisions for a single curvilinear incision from the angle of the mouth to the malar bone.‡

Liston performs this operation in the following manner:—"The extent of the disease is to be accurately ascertained, and

* Chelius, by South, vol. ii. p. 990.
† Principles of Surgery, third edition, p. 487.
‡ Cormack's Monthly Journal, February, 1843.

the points in which the bones require to be separated decided upon. If the os malæ be involved, and it is necessary to remove it, as well as the superior maxilla, a pair of straight tooth forceps, a full-sized bistoury, copper spatulæ, powerful scissors, artery forceps, and needles for interrupted and twisted suture, will be sufficient. If the superior maxilla only, with, perhaps, some of the smaller bones, is to be removed, then the addition to the apparatus of a small saw will be necessary, for the purpose of more readily effecting the separation of the os malæ from its anterior attachment. The proceeding is not to be dreaded on account of its extent; indeed, removal of the superior maxilla alone is the more troublesome. Supposing that the more extensive extirpation is required, incisions must be made so as to expose freely the tumor and bones where it is proposed to cut them. First of all, one of the central incisors must be extracted, either the one on the affected side or the other, according to the extent of the tumor. I have been obliged to remove a considerable portion of the jaw opposite to that principally affected, and in that case one of the molars was removed, in order to admit of the division of the bones. The point of the bistoury is entered over the external angular process of the frontal bone, is carried down through the cheek to the corner of the mouth, and is guided by the fore and middle fingers of the one or the other hand, as may be, placed in the cavity. A second incision, made along and down to the zygoma, falls into the other; then the knife is pushed through the integument to the nasal process of the maxilla, the cartilage of the ala is detached from the bone, and the lip is cut through in the mesial line. The flap thus formed is quickly dissected up and held by an assistant; the attachment of the soft parts to the floor of the orbit, the inferior oblique muscle, infra-orbital nerve, &c., are cut, and the contents of the cavity supported and protected by a bent copper spatula. The division of the bones is now undertaken; with the cutting forceps, the zygomatic arch, the junction of the os malæ and frontal bone by the transverse facial suture, and the nasal process of the superior maxilla, are cut in succession; then a notch having been cut out of the alveolar process, the palatine arch is clipped through by strong scissors, placed along it, one blade in the nostril of the affected side, the other in the mouth. Then it is that an assistant will be prepared to place his fingers on the trunk of one or both carotids. The tumor is now shaken from its bed, and as it is turned down, the remaining attachments are divided by the knife; the velum palati is carefully preserved, and also, if possible, the palatine plate of the palate bone;" or, as he states, the flap may be formed in the following way with less extensive cuts:—"The incisions were commenced at the inner canthus of the eye, carried by the side of and close to the ala of the nose, along the margin of the nostril, and then through the upper lip exactly in the middle line. Another incision was made from the commencement of the first, in a curved form, along the lower margin of the orbit, and of course in the direction of the fibres of the orbicularis palpebrarum. The flap thus formed was by dissection turned outwards, and held by an assistant until the processes were cut."*

Mr. Fergusson's directions for making the skin flap are, "that an incision should be made from the margin of the upper lip towards the nostril, and then from the ala, as high as within half an inch of the inner canthus of the eyelids; next the cheek should be laid open from the angle of the mouth (or near it,) as far as the zygomatic process of the malar bone, and if necessary, an incision at right angles with this one should extend from the external angular process of the frontal bone, towards the neck of the lower jaw; now the flap between the nose and the wound in the cheek should be dissected from off the tumor, and turned upwards on the brow; then that portion of the cheek below and behind the wound should be turned downwards, and the mucous membrane divided, so as to expose freely the interior of the mouth."† O'Shaughnessy, in his excellent Essay on Operations on the Jaws, has given the following interesting account of the removal of the upper jaw of a Hindu, of twenty-one years of age, which he performed in Nov. 1837:—An enor-

* Liston's Practical Surgery, p. 311, *et seq.*

† Practical Surgery, p. 548.

mous growth completely occupied the left side of the face, rising to a level with the floor of the orbit, and extending a long way below the inferior maxilla, but unattached to it; occupying the whole of the anterior and left side of the mouth, and protruding between the lips, pressing down the lower jaw, so as almost to make the chin touch the throat, and flattening the nose, so as to leave but little trace of the prominence of that organ. Still there was no difficulty of swallowing, and the patient seemed to breathe without inconvenience through the right nares. That portion of the tumor which protruded through the mouth was of a bright red color, and covered with mucous membrane, having at its upper part the canine and two incisors of its own side, with the central incisor of the opposite maxilla sticking out of it. The dimensions of this mass were as follows:—From the part near the ear to the most prominent part which protruded from the mouth, exactly twelve inches; and from that part which bulged below the inferior maxilla to the edge of the orbit, about ten inches. It looked, as near as may be, equal in size to the patient's head.

The principal source of pain to the patient appeared to be from distention and pressure on the surrounding parts. Notwithstanding its large size, the tumor seems to have been removed without much difficulty, the zygoma having been first cut through, and afterwards the malar bone, into the spheno-maxillary fissure, with Liston's bone-nippers. The orbital process of the superior maxillary bone and the nerve were next cut through with a strong knife, and afterwards the nasal process of the bone. The second incisive tooth on the opposite side having been drawn, the extent of the disease requiring it, the alveolar process and hard palate, as far back as the palatal process of the palate bone, were then cut through with the bone-nippers, and now all the strong attachments of the tumor being completely severed, he had no difficulty in removing that mass, carefully separating with the knife the palatal process of the superior maxilla from the palatal process of the palate bone, so as to preserve the soft palate from injury. The patient completely recovered.* M. Velpeau prefers an incision commencing at the commissure of the lips, and carried outwards and then upwards towards the temporal fossa. This incision may answer for a partial removal of the jaw, but will not expose the bone sufficiently for the section of its nasal process, for which purpose the somewhat vertical cut from the inner canthus down to the upper lip is very necessary. Dieffenbach has left the cheek alone, so as to preserve the branches of the portio dura, being content with an external incision through the upper lip, and along the back or prominent part of the nose, up towards the inner canthus, from whence he has carried the knife horizontally along the lower eyelid to the upper and outer part of the malar bone.

The practical point deducible from the opinions and experience of these eminent surgeons is, that it is by no means necessary to adhere to any particular line of incisions; a knowledge of anatomy, and the shape of the tumor, in short, the attendant circumstances of the case will modify them, and determine their course and extent.

In conclusion, there are a few points to which I wish specially to direct attention; and first, with reference to tying the carotid artery, as insisted on and put into practice by Lizars, in his operations on the jaw. Experience has proved that this proceeding is altogether unnecessary. The bleeding will be but trifling after once the flaps are formed, if the surgeon is not rash in the use of the knife; when detaching the tumor and bone from its posterior connections, the edge of the instrument should be kept close to the osseous tissue, and then the internal maxillary artery will not be endangered. All soft attachments should, if possible, be torn down with the finger, and the very depression and gentle wrenching of the mass from its bed with the forceps, will tend to lacerate the vessels entering from behind, and still further avert bleeding. It is an important object to prevent, as much as possible, the blood flowing towards

* On Diseases of the Jaws, with a brief Outline of their Surgical Anatomy, and a Description of the Operations for their Extirpation and Amputation. Calcutta, 1844.

the throat in the early part of the operation, hence the advantage of the sitting posture, and of beginning with the division of the cheek bone, before the nasal process of the upper jaw bone itself is attacked, as illustrated in my case.

In operations performed for the removal of either a portion or the whole of the superior maxillary bone, I do not conceive we can avail ourselves of the use of chloroform. I agree with Mr. Stanley, that there is a serious objection to its administration; for inasmuch as by its influence in annihilating sensibility, the irritability of the glottis is weakened, if not wholly lost, so there must be danger of a trickling of blood from the mouth into the glottis, without the excitement of a cough to expel it from the windpipe. The amount of this danger may be considered small, but it is sufficient to know that the apprehended evil has once occurred. Severe as the pain of these operations may be, it had better be endured than the risk of suffocation incurred, which must be regarded as a possible occurrence from the filling of the pulmonary air tubes and cells with blood. As to the division of the bone, cases will seldom occur where the chisel and mallet will be required; they cause great jarring, and, if possible, should not be used. So likewise may saws be dispensed with, for well-formed cutting pliers and powerful scissors, if the operator possesses the required strength to use them; and by the adoption of the latter, the section can be completed with such comparative rapidity that the sufferings of the patient are greatly diminished, and the shock abridged, while, at the same time be it remembered, if the instrument is steadily handled, the bone may be as evenly divided as by any other means, or, practically speaking, sufficiently so to permit healthy repair of the cut edges, a fact very remarkably exemplified in the case of the young man I operated on.

## Figures and Tables

**Fig. 1. f1:**
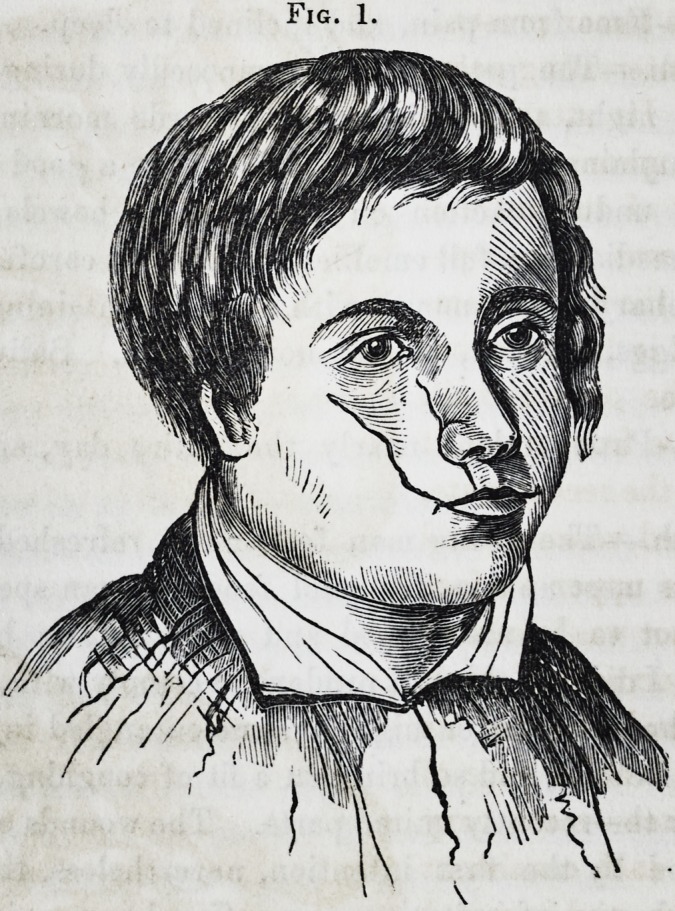


**Fig. 2. f2:**
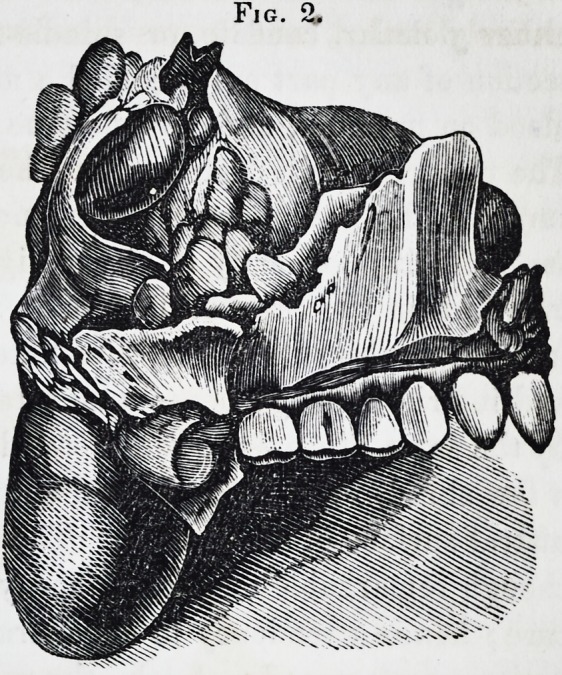


**Fig. 3. f3:**